# Revisiting future extreme precipitation trends in the Mediterranean

**DOI:** 10.1016/j.wace.2021.100380

**Published:** 2021-12

**Authors:** George Zittis, Adriana Bruggeman, Jos Lelieveld

**Affiliations:** a Climate and Atmosphere Research Center, The Cyprus Institute, Nicosia, Cyprus; b Energy, Environment and Water Research Center, The Cyprus Institute, Nicosia, Cyprus; c Dept. of Atmospheric Chemistry, Max Planck Institute for Chemistry, Mainz, Germany

**Keywords:** Climate change, Mediterranean, Extreme precipitation, Trends, CORDEX

## Abstract

Global warming is anticipated to intensify the hydrological cycle. However, this is neither expected to be globally uniform nor is the relationship between temperature increase and rainfall intensities expected to be linear. The objective of this study is to assess changes in annual rainfall extremes, total annual precipitation, and their relationship in the larger Mediterranean region. We use an up-to-date ensemble of 33 regional climate simulations from the EURO-CORDEX initiative at 0.11° resolution. We analyse the significance of trends for 1951–2000 and 2001–2100 under a ‘business-as-usual’ pathway (RCP8.5). Our future projections indicate a strong north/south Mediterranean gradient, with significant, decreasing trends in the magnitude of daily precipitation extremes in the south and the Maghreb region (up to −10 mm/decade) and less profound, increasing trends in the north. Despite the contrasting future trends, the 50-year daily precipitation extremes are projected to strongly increase (up to 100%) throughout the region. The 100-year extremes, derived with traditional extreme value approaches from the 1951–2000 simulations, underestimate the magnitude of these extreme events in the 2001–2100 projections by 30% for the drier areas of the Mediterranean (200–500 mm average annual rainfall) and by up to 20–30% for the wetter parts of the region. These 100-year extremes can occur at any time in any Mediterranean location. The contribution of the wettest day per year to the annual total precipitation is expected to increase (5–30%) throughout the region. The projected increase in extremes and the strong reductions in mean annual precipitation in the drier, southern and eastern Mediterranean will amplify the challenges for water resource management.

## 1 Introduction

Recent accelerated warming, which exceeds global rates, is exacerbating environmental stresses in the Mediterranean region (Cramer et al., 2018). According to future projections, the Mediterranean climate change hot-spot will continue to warm (Zittis et al., 2019; Cherif et al. 2020). The anticipated warming differs by season and depends on greenhouse gas emission pathways. The warming will likely coincide with changes in the hydrological cycle, mainly precipitation decreases and increased manifestation of droughts (Spinoni et al., 2020; Zittis et al., 2019). The level of future warming is characterized by high robustness and significance both in global and regional model projections (Lelieveld et al., 2016; Zittis et al., 2019). On the other hand, the inter-model agreement and robustness of precipitation projections are much lower. For the Mediterranean, the spread of climate models is comparable or sometimes larger than the climate change signal (Lelieveld et al., 2016; Zittis et al., 2019). Individual models can even disagree on the sign of projected changes for particular locations or aspects of precipitation, such as seasonality (Giorgi et al., 2016; Rajczak and Schär, 2017).

Besides the uncertainties involved in the precipitation projections, increases in extreme rainfall events are expected, since, in a warmer world, the hydrological cycle will intensify (Allen and Ingram, 2002; Giorgi et al., 2019). Several studies have highlighted that anthropogenic climate change can affect the severity and frequency of high-impact extreme precipitation events in the region. A significant intensification of daily precipitation extremes for the mid- and high-latitudes, including the Mediterranean, is projected by the end of the century (Toreti et al., 2013). Increases in both dryness and wetness indicators, implying increased probabilities of both drought and flooding events, are will likely occur even though there will be less total precipitation in the Mediterranean (Chen et al., 2014). Statistically significant increases in annual daily extremes are expected by the end of the 21st century for 25 global models under the RCP8.5 emission pathway, whereas for arid regions, less than half of the models showed significant increases, and three models showed significant decreases (Donat et al., 2019). However, the coarse spatial resolution of the global models used in these studies does not allow the representation of smaller-scale processes that affect the generation of rainfall and smooths out extremes.

From analysing an ensemble of Mediterranean projections, Drobinski et al. (2018) proposed a hook-shaped relationship between temperature and precipitation extremes, with a negative slope at high temperatures and a slope following Clausius–Clapeyron scaling at low temperatures. Overall, their ensemble predicted more intense precipitation extremes in the future, but their conclusions were based on a limited number of climate projections. Both global and regional models project the historical 1-in-100-year maximum precipitation to become more frequent for parts of southern Europe, with up to a tenfold frequency increase for some regions (Martel et al., 2020). Several recent studies based on global and regional climate projections have identified a north-south gradient, with projected increases of extreme precipitation indicators in the northern Mediterranean and decreases in the south (Lionello and Scarascia, 2020; Tramblay and Somot, 2018). The latitude-dependent response of precipitation changes to global warming has also been identified by Thackeray et al. (2018). They suggest that parts of the Mediterranean lie in the transitional zone where, on the one hand, decreases of weak-moderate precipitation are expected, while on the other hand, climate models suggest an increase in extremes.

The changes in precipitation extremes challenge the assumption of climate stationarity, which forms the basis of extreme value analysis, as used for the planning and design of infrastructure (e.g., Salas and Obeysekera, 2014). The use of time as a covariate in the fitting of the extreme value distributions functions has gained attention as a practice for dealing with the transient nature of a changing climate in hydrologic and hydraulic design (e.g., Cheng et al., 2014). However, Serinaldi and Kilsby (2015) pointed out that these time-varying models may not remain valid for the full design lifetime of the intended infrastructure. A combination of an ensemble of climate simulations, regional analysis (spatial pooling), and temperature scaling could be used to project future extremes over most of Northern America (Li et al., 2019). Nevertheless, this approach may not apply at all latitudes or under all climate regimes.

Although there is a significant number of studies on the effects of global warming on future Mediterranean precipitation extremes, most of the literature is either based on the results of global climate projections, which are known to underestimate precipitation extremes, or on individual regional models or multi-model output of a limited number of ensemble members, which is a shortcoming in view of the large spatial and temporal variability associated with precipitation. Despite the different local and regional responses to climate change, the Mediterranean and southern Europe are often treated as one sub-region. That approach can smooth the magnitude of projected changes and lead to erroneous conclusions. Moreover, large parts of the basin, such as the southeast, often receive less attention. The selection of sub-periods of limited duration (for example, 20 or 30 years) may not suffice to represent the statistics of extreme events. Within this framework, we aim at addressing some of these limitations.

Based on an ensemble of 33 regional climate simulations and robust non-parametric statistics, the main objectives of the present study are: (i) to assess the direction, significance and inter-model agreement of the 21st-century trends of annual daily precipitation extremes (RX1day) in the larger Mediterranean region, (ii) to investigate changes in 50-year and 100-year extremes between the recent past (1950–2000) and the 21st century, (iii) to approximate the time of occurrence of 100-year extremes during the 21st century, and (iv) to assess changes in total annual precipitation and the contribution of daily extremes to the annual total precipitation budget. For assessing the differential effect of global warming on precipitation extremes in a region marked by many and steep spatial gradients in average annual precipitation, we subdivide the area into five precipitation classes. Considering that the impact of precipitation extremes is often relatively large in urban environments, we also investigate the changes in extremes for 32 Mediterranean cities.

## 2 Data and methods

### 2.1 Description of simulations and data

The present analysis is based on the high-resolution version of the most comprehensive set of regional climate simulations, available at the time of writing. This is the European (EURO) initiative of the Coordinated Regional Climate Downscaling Experiment (CORDEX). This version of EURO-CORDEX simulations (Jacob et al., 2020) has been extensively compared against observations and was found to improve several aspects of extreme rainfall compared to lower resolution regional and global experiments (Kotlarski et al., 2014; Casanueva et al., 2016; Prein et al., 2016; Soares and Cardoso 2018; Fantini et al., 2018; Iles et al., 2020). In view of the available evidence in recent publications, we did not perform additional evaluation by comparing with observations.

**Fig. 1:**
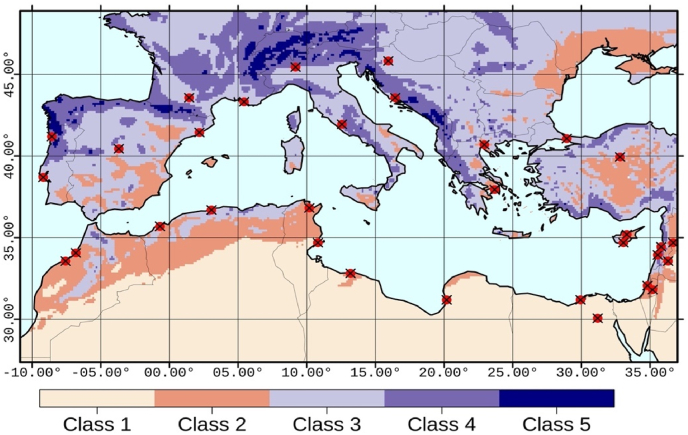
Extent of the Mediterranean region considered in the present study and subdivision into precipitation classes according to the 1951–2000 simulated (EURO-CORDEX ensemble mean) annual precipitation climatology (Class 1: 0–200 mm/year, Class 2: 200–500 mm/year, Class 3: 500–1000 mm/year, Class 4: 1000–2000 mm/year, Class 5: >2000 mm/year). Locations of the analysed 32 Mediterranean cities are indicated with red points. (For interpretation of the references to colour in this figure legend, the reader is referred to the Web version of this article.)

We considered all publicly available EURO-CORDEX simulations, at the time of writing, with a horizontal spatial resolution of 0.11° (~12 km). Then we took a subset that only included experiments with daily precipitation data series available from 1951 until the end of the 21st century. For example, a number of simulations that start after the year 1970 were excluded. We analysed a ‘high forcing’ or ‘business-as-usual’ Representative Concentration Pathway (RCP8.5), being consistent with the observed global and regional warming trends (Zittis et al., 2019). Our final ensemble consisted of 33 simulations (Table S1 of the Supplementary Material) and is based on a combination of six global Earth system models and nine regional climate models (RCMs). The global models that provided the initial and boundary conditions for the dynamical downscaling were part of Phase 5 of the Coupled Model Intercomparison Project (CMIP5). The different combinations of CMIP5/CORDEX models included in this study are all considered to give equally likely projections, in the sense of ‘one model, one vote’. From the extended European domain, we extracted data only for the Mediterranean region as defined in Fig. 1.

To analyse regional differences in the trends of annual daily extremes in the 21st century, we divided the region into five classes or regimes according to the simulated (ensemble mean) annual precipitation amounts for the reference period 1951–2000 (Fig. 1). The arid (0–200 mm/year) and semi-arid (200–500 mm/year) regimes are included in Classes 1 and 2 respectively. Class 3 is defined as the more temperate regions of annual precipitation between 500 and 1000 mm/year. Class 4 includes wetter areas (1000–2000 mm/year), mainly found in the northern part of the Mediterranean region. Finally, Class 5 includes the very wet mountainous and coastal areas, where the simulated annual precipitation exceeds 2000 mm. The percentages of total land grid cells that fall in each class are 34.3%, 15.4%, 34.6%, 14%, and 1.7%, respectively.

The impacts of extreme precipitation and related flooding events are often more devastating in urban than in rural environments. Therefore, we extracted precipitation extremes and totals for 32 capital cities and large urban centers in the Mediterranean region from the nearest land model grid cell of all 33 models. The total human population of the selected cities is currently nearly 100 million, or about 20% of the Mediterranean region population.

### 2.2 Precipitation indices

From each EURO-CORDEX simulation, we extracted the annual time-series of daily precipitation extremes (Rx1day). This is defined as the highest value of daily rainfall during a year. Thus, our Rx1day time-series for each model and each grid-point consists of 150 values, one for each year. Exceptions are the regional simulations driven by Had-GEM2-ES global earth system model, which end in the year 2099.

For the assessment of future trends and changes in mean climatic conditions, we calculated the annual total precipitation (PRCPTOT) for each year. Moreover, we introduce a simple yet descriptive metric, defined as the ratio between the maximum daily extreme and the annual total precipitation for each year:

(1) RxTRatio=Rx1dayPRCPTOT

The RxTRatio describes the contribution of the wettest day per year to the total annual precipitation. This index could be of particular interest for the arid and semi-arid parts of southern Mediterranean. In such climatic regimes, a significant proportion of annual rainfall can come from single extreme events that occur within a short period.

All three indices (Rx1day, PRCPTOT, RxTRatio) were calculated for each model experiment and grid-cell separately. They were averaged per grid-cell for all 33 members to visualize climatologies.

### 2.3 Trend analysis

To investigate the statistical significance of the 21st-century trends (2001–2100) for Rx1day, PRCPTOT, and RxTRatio, we applied the non-parametric Mann-Kendall test (Mann, 1945). We consider significant trends to have p-values lower than 0.05. For assessing the direction and magnitude of Rx1day trends, we used the Sen's Slope Estimator. This is a non-parametric test based on Kendall's Tau statistic (Sen, 1968). Sen's Slope is defined as the median of all pairs of slopes (*Q*) of the *N* pairs of a time series and is calculated as follows:

(2) $$Q_{i}=\frac{X_{j}-X_{k}}{j-k}\;\text{for}\;i=1,\;\ldots ,\;N,$$

where *X*_*j*_ and *X*_*k*_ are the data values at times *j* and *k* (*j* > *k*), respectively.

### 2.4 Changes in 50-year extremes and significance of projected changes

To analyse precipitation changes, we defined as a control reference period (CTL) the 50-year period of 1951–2000. For the projected changes, we defined two 50-year periods. One for each half of the 21st century (21C1: 2001–2050 and 21C2: 2051–2100). For each 50-year period, we extracted the absolute maxima of the RX1day values of all grid cells and models. This is the daily rainfall with a return period of 50 years, referred to as the 50-year extreme. These extremes were subsequently averaged over all 33 model simulations, for each grid cell.

To assess the statistical significance of projected changes, we applied the non-parametric one sample Wilcoxon signed-rank test (Woolson, 2005), considering the confidence level of 99%. Here we assumed that the EURO-CORDEX ensemble set (33 models) represents a random sample of future projections. We test the changes between each sub-period (21C1, 21C2) and the control period. The null hypothesis is that the change in the sample median is zero. The Wilcoxon rank test was applied for the changes in the three precipitation indices (Rx1day, PRCPTOT, RxTRatio) on a grid cell basis.

### 2.5 Extreme value analysis and changes in 100-year extremes

To analyse changes in 100-year extremes between the recent past (1951–2000) and the 21st-century future, we first tested the CTL period for time trends, using the Mann-Kendall test. In case significant time trends are absent, generalized extreme value (GEV) distribution functions can be fitted through the annual Rx1day values for estimating the 100-year rainfall extremes. For the future period, the 100-year extremes were derived as the absolute maxima of the RX1day time series for 2001–2100.

We applied the commonly used generalized extreme value (GEV) distribution (Jenkinson, 1955), defined as:

(3) $$F\left(x;\mu ,\;\sigma ,\;\xi \right)=\;\left\{ \begin{array}{l} exp\left[-exp\left(-\frac{x-\mu }{\sigma }\right)\right],\;\xi =0,\;\\ exp\left[-{\left(1+\xi \frac{x-\mu }{\sigma }\right)}^{-1/\xi }\right],\;\xi \neq 0,\;1+\xi \frac{x-\mu }{\sigma }>0\; \end{array}\right.$$

where *μ* is the location parameter, *σ* is the (positive) scale parameter, and *ξ* is the shape parameter. GEV is appropriate for modeling block maxima (for large blocks, such as annual precipitation maxima). It is often called a family of distribution functions since it encompasses the three types of Extreme Value Distributions: Gumbel (*ξ* = 0, light tail), Frechet (*ξ* > 0, heavy tail), and the reverse Weibull (*ξ* < 0, bounded upper tail). For the estimation of the GEV parameters, we used the Maximum Likelihood (ML) technique (Prescott and Walden, 1980). This approach is reliable for time-series of rainfall between 30 and 50 years (Kharin et al., 2013), which is the case for the sub-period length in our analysis. The GEV-ML estimation was applied to the CTL period (1951–2000) to approximate the 1-in-100-year extreme daily precipitation values. Subsequently, we compared these with the projection-derived 1-in-100-year extremes for the 21st century. These are defined by the absolute maxima of Rx1day for 2001–2100.

We calculated the 95% confidence intervals (CIs) of the GEV-ML distribution for selected grid points (representative Mediterranean cities) for the model with the local median Sen's slopes of the Rx1day values. The confidence intervals were estimated using parametric bootstrapping with 1000 iterations (Kyselý, 2010).

## 3 Results

### 3.1 Trends in precipitation extremes and totals in the 21st century

The analysis of the 21st-century trends of annual Rx1day reveals considerable inter-model variance in terms of the sign, magnitude, and level of statistical significance (Table 1 and Fig. S1 of the Supplementary Material). For example, in the different EURO-CORDEX experiments, the percentage of the total number of land grid-cells where significant future trends are found ranges from 1% to 46% for negative and 5%–28% for positive trends (Table 1). This variance between the climate simulations is, for most cases, not systematic in the sense that there is no evident dependency on the global or regional model selection. Some exceptions include the lack of significant negative trends for the arid regions (Class 1–2) for the projections driven by the global CNRM-CM5 and EC-EARTH models (Simulation IDs 1–11) and the larger fraction of cells with significant negative trends for these regions for the cluster of projections driven by IPSL-CM5A-MR (Simulation IDs 12–15, Table 1). Moreover, the inter-model range is highest for the two low rainfall classes (Class 1 and 2) but very similar for the three wettest classes. This indicates more uncertainty for the drier areas.

**Table 1:** Percentage of grid cells with statistically significant negative (left) and positive (right) trends of annual maximum daily precipitation (Rx1day) projected for the 21st century. Results are presented for all grid cells and for 5 precipitation classes (Class 1: 0–200 mm/year, Class 2: 200–500 mm/year, Class 3: 500–1000 mm/year, Class 4: 1000–2000 mm/year, and Class 5: >2000 mm/year). Horizontal lines separate regional simulations driven by different global climate models (see Table S1 Supplement).

**% of grid cells with significant negative trends**							**% of grid cells with significant positive trends**						
**Sim. ID**	**All grid cells**	**Class 1**	**Class 2**	**Class 3**	**Class 4**	**Class 5**	**Sim. ID**	**All grid cells**	**Class 1**	**Class 2**	**Class 3**	**Class 4**	**Class 5**
**1**	2.7	4.8	4.1	1	0.3	0.1	**1**	18.5	0.4	13	30.2	37.3	37.1
**2**	1	2.3	0.5	0.2	0.3	0	**2**	15.8	2.2	16.6	23.1	27.6	36.5
**3**	3.6	6	6.5	1.3	0.6	0.7	**3**	19.3	0	12.8	33.9	34.4	45.6
**4**	0.9	0.2	1.2	1.3	1.7	0.3	**4**	15.5	5.1	12.8	25.8	17.3	28
**5**	0.9	1.1	1.6	0.7	0.3	0	**5**	20	1.7	20.4	32.7	30.4	38.7
**6**	1.9	1.9	4.6	1.5	0.1	0	**6**	15.3	2.4	16.6	20.7	29.7	36.6
**7**	3.6	9.1	1.5	0.7	0.2	0	**7**	15.9	0.1	13.1	24.1	33.7	44
**8**	5.9	14.2	3.6	1.1	0.3	0.4	**8**	12.8	0.1	9.6	19.2	27.8	43.3
**9**	1	0.9	2.2	0.7	0.5	0	**9**	8.3	6.1	3.5	9.1	14.3	25.9
**10**	9.5	24.3	3.7	1.3	1.1	0.9	**10**	14.8	0	11.2	27.6	23.7	13.3
**11**	5.8	14.7	2.8	0.7	0.4	1.2	**11**	13.7	0.2	12.6	25.2	20.5	8.6
**12**	42.6	93.7	38.8	8.8	9.6	4.2	**12**	10.7	0	3.5	18.7	24.1	17.6
**13**	28.2	60.7	31.4	5.9	3.3	1.7	**13**	28.3	0	19.5	48.2	53.9	61.3
**14**	31.3	68.1	27.3	8.3	5.9	4.3	**14**	11.6	0	1.9	18.9	31.4	18.9
**15**	45.6	95.7	44.8	12.3	11	6.4	**15**	9.5	0	0.2	18.1	21.6	12.6
**16**	27.2	65.9	19.9	4	1	0	**16**	16.3	0	11.6	26	33.6	49.3
**17**	31.4	73	27.8	5.6	1.2	0.3	**17**	13.6	0	15.9	20.5	24.6	35.3
**18**	25.5	58.3	22.5	5.1	1.9	1.7	**18**	19.7	0	15.1	36.2	32.4	20.9
**19**	8.2	14.7	10.2	4	1.2	0	**19**	28.3	3.1	24.6	47.6	45.5	32.3
**20**	11.9	27.3	10.7	2.3	1.1	0	**20**	24.2	0.2	19.8	44	39.7	17.2
**21**	4.1	3.8	11.5	2.7	0.5	0	**21**	22.6	5.7	20.4	35.1	33.1	42.4
**22**	22.9	56.8	17.8	2	0.3	0.4	**22**	11.3	0.1	3.5	18.9	26.7	26.3
**23**	21	48.9	22.1	2.1	0.9	1.1	**23**	10.4	0	9.9	15.7	21.3	26.5
**24**	24.2	57.2	19	3.4	3.1	1.1	**24**	13.3	0	6.9	23	28	21.7
**25**	17.7	42.8	13	2.4	0.9	1.6	**25**	13.4	0.3	9.6	21.3	30.3	13.3
**26**	8.5	15.8	13.1	2.8	0.6	1.1	**26**	11.2	0.1	7.6	16	27.5	32.6
**27**	9.2	17.1	12.4	2.6	2.8	5.6	**27**	9.8	0	3.8	17.8	20.3	11.8
**28**	28.6	64.9	23.3	6.9	2.4	3.4	**28**	5.3	0	1.5	7.9	13.4	25.7
**29**	24.1	51	28.1	5.3	2.8	2.7	**29**	12.7	0	8	23.3	24	2.9
**30**	4.6	4.3	12.1	3.1	1.8	0.4	**30**	10	0.6	6.7	17.9	16.8	13
**31**	11.7	21.7	15.8	4.1	2.3	2.7	**31**	13.3	0.1	10.5	26.4	17.8	0.4
**32**	5.7	4.5	14.1	5	1.5	0.1	**32**	7.4	0.9	5.3	9.8	16.5	30.8
**33**	14.1	25.3	19.7	4.6	4.8	6	**33**	10.6	0.4	7.7	20.4	15.1	6.4
**Ens. Mean**	14.7	31.8	14.8	3.4	2	1.5	**Ens. Mean**	14.6	0.9	10.8	24.3	27.1	26.6
**Ens. Median**	9.5	21.7	13	2.7	1.1	0.7	**Ens. Median**	13.4	0.1	10.5	23	27.5	26.3
**Ens. Min.**	0.9	0.2	0.5	0.2	0.1	0	**Ens. Min.**	5.3	0	0.2	7.9	13.4	0.4
**Ens. Max.**	45.6	95.7	44.8	12.3	11	6.4	**Ens. Max.**	28.3	6.1	24.6	48.2	53.9	61.3

**Fig. 2:**
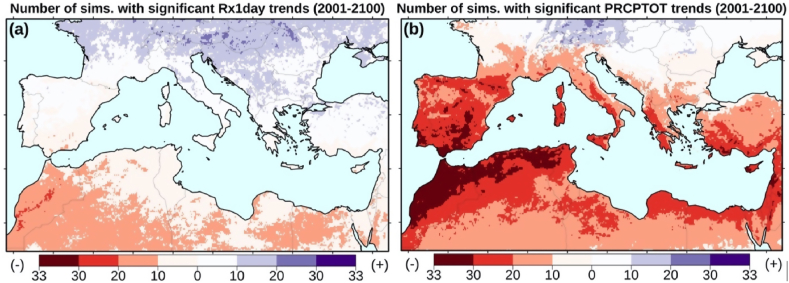
Number of model simulations with significant (p-value < 0.05) positive (blue) and negative (red) non-parametric trends of daily precipitation extremes (Rx1day, left panel) annual precipitation (PRCPTOT, right panel) for the 21st century. (For interpretation of the references to colour in this figure legend, the reader is referred to the Web version of this article.)

Despite the relatively strong variance and considering the large size of our ensemble, useful conclusions can still be drawn. The mean percentage of grid cells with significant trends (regardless of the sign) is nearly 30%. Moreover, there is a distinct north-south gradient in the direction of future daily precipitation extremes (Fig. 2a). Statistically significant negative trends are expected for the drier, southern Mediterranean territories, including south Spain. On the other hand, large parts of the northern regions show significant positive trends. Strong inter-model agreement (>66%) is only evident in limited parts of North Africa and the Maghreb region. Furthermore, there is a transitional zone at latitudes around 35–40°N (indicated by the white areas in Fig. 2a) where less than ten models produced either significantly positive or negative trends, indicating low statistical significance and/or low inter-model agreement in the direction of trends.

**Fig. 3:**
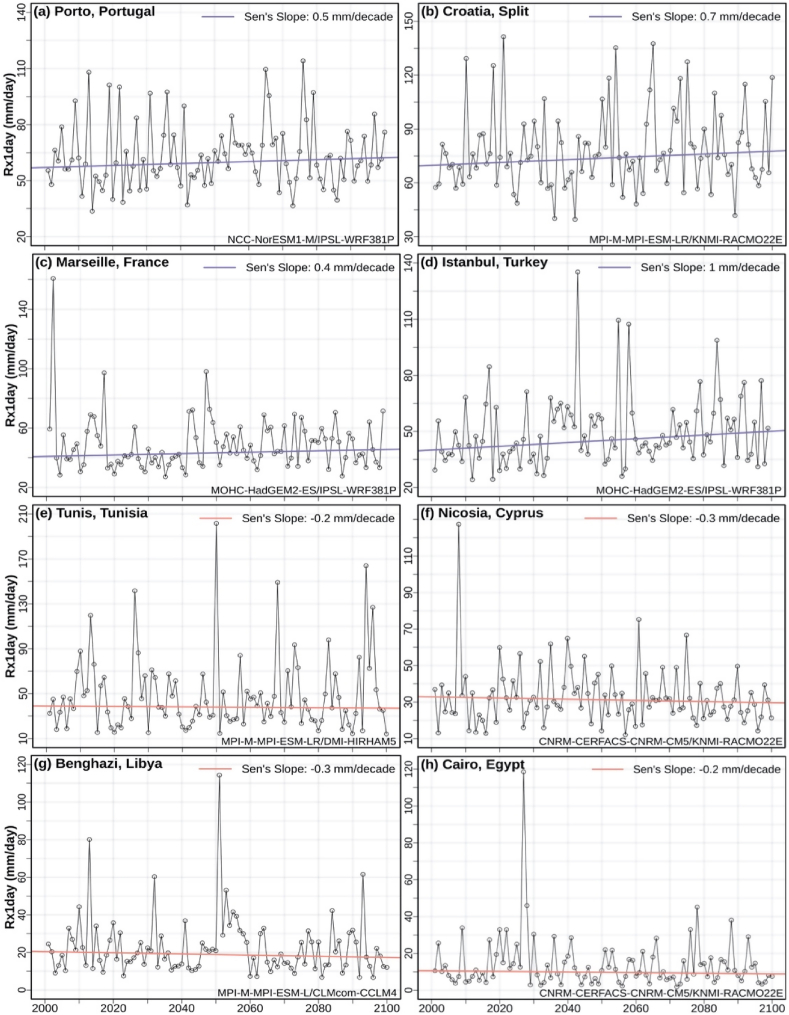
Time-series of annual maximum daily precipitation (Rx1day) and Sen's Slopes for the 21st century of the local median EURO-CORDEX model simulation, for eight Mediterranean cities. Blue lines indicate positive trends and red lines indicate negative trends. (For interpretation of the references to colour in this figure legend, the reader is referred to the Web version of this article.)

Time-series of Rx1day values for eight Mediterranean cities (representative for precipitation classes 1–4) are indicatively presented in Fig. 3 for the simulation with the local median Sen's Slope. The model combination of local median Sen's Slope (also indicated in the panels of Fig. 3) appears to be different for the most of the selected locations. A positive slope of near 1 mm/decade is found for Istanbul and Split (precipitation classes 3 and 4 respectively), whereas small negative slopes (−0.2 to −0.3 mm/decade) are found for the southern Mediterranean cities of Tunis, Nicosia, Benghazi, and Cairo that are simulated to receive annual precipitation less than 500 mm/year (precipitation classes 1–2).

According to the Mann-Kendall test results (Fig. 2b), significant negative trends of annual total precipitation (PRCPTOT) prevail for the 21st century (2001–2100). Except for some continental regions in the central and eastern European part of the domain, the EURO-CORDEX models suggest declining future precipitation trends (Fig. 2b). Overall, there is a convincing inter-model agreement (greater 66% of the 33 models) towards statistically significant negative trends. This is evident in extensive areas along the Mediterranean coast, including the Maghreb region, the Iberian Peninsula, western Greece, the Levantine, southern Anatolia, and other areas.

### 3.2 Changes in 50-year precipitation extremes and totals

**Fig. 4:**
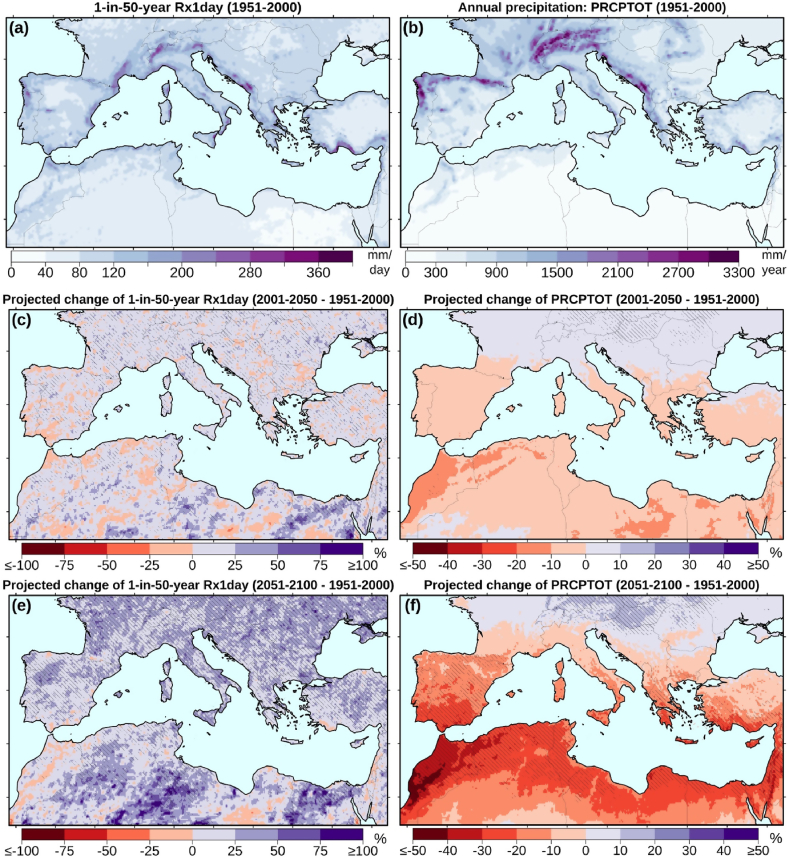
Simulated climatology of maximum daily precipitation (Rx1day, left panels) and total annual precipitation (PRCPTOT, right panels) for the 1951–2000 reference period (mean of 33 EURO-CORDEX simulations) (a, b), and projected changes for two future periods (e–f). Hatching (panels c–f) denotes statistically significant changes at the 99% confidence level.

Simulated (1951–2000) ensemble mean values of the 50-year maximum daily precipitation (Rx1day) are presented in Fig. 4a. According to the EURO-CORDEX simulations, the maximum 50-year extremes (up to 300 mm/day or more) are simulated in mountainous regions (for example, the Alps and the Pyrenees) and elevated areas near the coasts (for example, Galicia at the Atlantic coast of Spain and the Dalmatian coast at the Adriatic Sea). In such regions, the combination of humid air masses and topography can favour the conditions for extreme precipitation development. The simulated Rx1day values for 32 Mediterranean cities are presented in Table 2.

**Table 2:** Projected 50-year daily precipitation extreme (mm/d), mean annual precipitation (PRCPTOT, mm/yr), and extreme to total precipitation ratio (RxTRatio, %), for 1951–2000 (CTL), 2001–2050 (21C1) and 2051–2100 (21C2) periods for 32 Mediterranean cities.

	**City**	**Country**	**Precip. Class**	**Rx1day (maximum)**			**PRCPTOT (mean)**			**% RxTRatio (mean)**		
				**CTL**	**21C1**	**21C2**	**CTL**	**21C1**	**21C2**	**CTL**	**21C1**	**21C2**
1.	Cairo	Egypt	1	38	51	57	48	44	37	25	28	32
2.	Alexandria	Egypt	2	79	74	77	213	197	166	14	15	17
3.	Tripoli	Libya	2	105	113	123	254	235	204	14	16	19
4.	Benghazi	Libya	1	64	76	86	163	148	120	16	17	21
5.	Tunis	Tunisia	2	116	132	156	367	347	301	11	12	14
6.	Sfax	Tunisia	2	125	128	180	212	200	179	17	18	20
7.	Algiers	Algeria	3	157	145	153	644	593	490	9	10	12
8.	Oran	Algeria	2	125	125	125	442	401	317	11	11	14
9.	Casablanca	Morrocco	2	73	81	92	379	332	246	10	12	15
10.	Rabat	Morrocco	3	106	108	107	588	508	381	9	10	13
11.	Madrid	Spain	3	90	88	97	566	538	470	7	7	9
12.	Barcelona	Spain	3	154	164	190	591	604	558	10	11	12
13.	Lisbon	Portugal	3	135	130	148	701	661	570	8	9	11
14.	Porto	Portugal	4	182	181	204	1741	1686	1534	5	6	7
15.	Marseille	France	3	149	150	184	646	654	604	9	9	11
16.	Toulouse	France	3	100	109	124	841	833	785	5	5	6
17.	Rome	Italy	3	113	124	154	776	785	742	6	7	8
18.	Milan	Italy	4	123	152	166	1021	1054	997	6	7	8
19.	Zagreb	Croatia	3	93	111	120	832	879	902	5	6	6
20.	Split	Croatia	4	150	178	186	1299	1340	1326	6	6	7
21.	Athens	Greece	2	114	112	114	379	360	328	11	12	14
22.	Thessaloniki	Greece	2	93	109	132	436	428	407	9	10	11
23.	Ankara	Turkey	3	61	64	80	510	511	477	6	6	7
24.	Istanbul	Turkey	3	102	115	158	673	686	664	7	7	8
25.	Nicosia	Cyprus	2	79	96	123	264	254	213	11	13	15
26.	Limassol	Cyprus	2	104	127	122	382	362	290	12	13	15
27.	Damascus	Syria	2	72	69	81	222	206	166	13	15	18
28.	Homs	Syria	2	60	60	67	221	211	177	11	12	14
29.	Beirut	Lebanon	4	148	162	154	1083	1048	877	8	8	10
30.	Tripoli	Lebanon	3	119	119	134	687	671	572	9	9	11
31.	Jerusalem	Israel	2	76	79	78	290	264	201	12	14	17
32.	Tel-Aviv	Israel	2	126	129	124	499	465	369	12	13	15

The projected changes of the 50-year extremes for periods 21C1 and 21C2 (with respect to the CTL period) are presented in Fig. 4(c–f). For the first half of the 21st century (21C1), positive changes (up to 25%) prevail throughout the Mediterranean (Fig. 4c), nevertheless, these changes are statistically significant for the 33-member ensemble only for about one-fifth of the grid cells. This is the case even in southern Mediterranean regions, where projections indicate decreases in total precipitation or negative Rx1day trends for the 21st century (Fig. 2, Fig. 4d). The projected changes in the 50-year daily precipitation extremes are expected to be larger in the second half of the 21st century (Fig. 4e), irrespective of the expected profound drying of the region, as shown by the climatic means of total precipitation (Fig. 4d). In more than half of the land grid cells in the Mediterranean (56%), these changes (mainly increases) are statistically significant (99% confidence level). Under this ‘business-as-usual’ scenario, for many grid cells throughout the Mediterranean, the increase of 50-year extremes will likely reach 100% with respect to the CTL period.

This could potentially be associated with unprecedented flooding events. unprecedented flooding events. Such conditions will be associated with significant ecological and societal impacts even in the arid southern parts of the Mediterranean (including parts of the Sahara Desert, Egypt, coasts of Libya, and Tunisia). For example, under this ‘business-as-usual’ scenario, daily precipitation events of 100 mm could occur in areas where the CTL 50-year maxima did not exceed 50 mm/day. Since parts of the above-mentioned regions are densely populated, several cities and large urban centers will be affected (Table 2). Further, in the much wetter parts of southern Europe, Rx1day increases in the order of 25–50% can be expected. For example, in cities such as Milan and Barcelona, the 50-year rainfall extremes are projected to increase from 120 to 170 mm/day and from 150 to 190 mm/day, respectively.

The multi-model ensemble mean climatology of the simulated (1951–2000) total annual precipitation (PRCPTOT) is presented in Fig. 4b. This figure illustrates the strong climatic gradient across the Mediterranean. For most of the northern part of the basin, the simulated annual precipitation exceeds 1000 mm/year. Locally, in regions of high elevation, windward slopes and coastal areas, this is found to reach 3000 mm/year. On the other hand, in the arid and semi-arid parts of North Africa and the Middle East annual precipitation is limited to below 200 mm/year. The simulated PRCPTOT values for 32 Mediterranean cities are presented in Table 2. For the first half of the 21st century (Fig. 4d), the EURO-CORDEX projections suggest minor changes (±10%). However, for most of the Mediterranean, a decreasing tendency is more distinct. For this sub-period (21C1), the projected changes are mostly not statistically significant, with the exception of the precipitation increases in the northern part of the domain. For the second half of the 21st century (Fig. 4f), stronger annual precipitation decreases (20–40%) are expected on average for most of the region and particularly in the latitudinal zone between 30 and 45°N. Conversely, in northern regions, mean annual precipitation is expected to increase by 10–20%. The projected changes are statistically significant (99% confidence level) in about one-third of the region (30% of the grid cells). This is the case for most of the southern and eastern Mediterranean coast, Iberia, southern Greece and Anatolia that will likely experience drying, and for parts of central Europe, which are expected to become wetter.

**Fig. 5:**
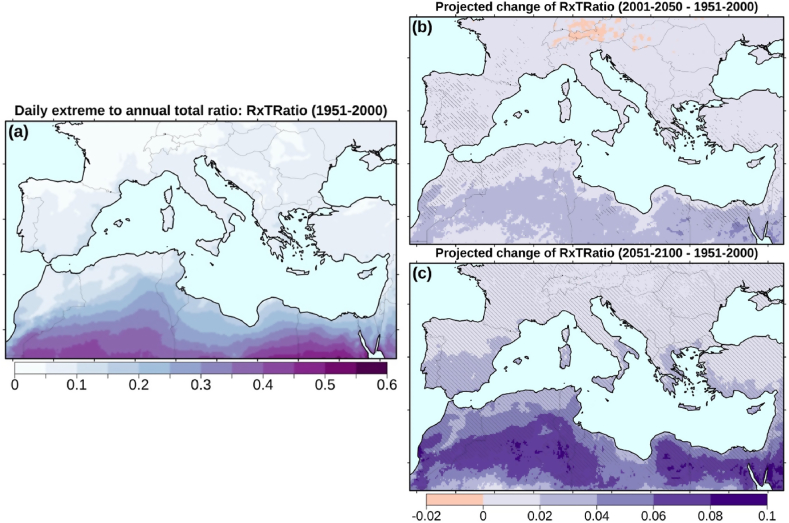
Simulated climatology of the daily extreme (Rx1day) to annual total (PRCPTOT) precipitation ratio (RxTRatio) for the reference period (a) and projected mean changes for two future periods (b, c). Hatching in the right panels denotes statistically significant changes at the 99% confidence level.

The contribution of the wettest day of the year to the annual precipitation total is given by the RxTRatio (Fig. 5). The simulated climatology of this index for the CTL period (ensemble mean) is presented in Fig. 5a. The ratio is higher for the dry areas of North Africa, where according to the EURO-CORDEX simulations about one to two-thirds of annual precipitation are on average the result of single-day events. For example, for the city of Cairo in Egypt, about 25% of the annual total precipitation is on average attributed to a single-day event (Table 2). Most of the CORDEX simulations indicate positive RxTRatio trends for the 21st century (not shown), while these are found to be statistically significant mainly in Mediterranean mid-latitudes (33–43°N). The projected RxTRatio changes are presented in the right panels of Fig. 5. Positive changes are evident throughout the region. For 2001–2050 increases are limited to 0–0.04 (or 10–20%) and are mostly not statistically significant. However, for the second half of the 21st century, RxTRatio increases are projected to exceed 0.04–0.06 (or 30–40%) in many regions. These increases are statistically significant at the 99% confidence level in about 80% of the land grid cells (Fig. 5c). For the Mediterranean mid-latitudes, this could be the result of the combined decreasing PRCPTOT and the increasing Rx1day values. For the southern Mediterranean and the Maghreb region, where the projected increase of RxTRatio is stronger (increases up to 50–60%), Rx1day and PRCPTOT are both expected to decrease (Fig. 2, Fig. 3). This is an indication that Rx1day will likely decrease at slower rates than PRCPTOT. Additional challenges on water resources management are expected if most of the precipitation budget results from a few events per year. The vulnerable water-stressed communities of the region will be most affected.

### 3.3 Changes in 100-year extremes

**Fig. 6:**
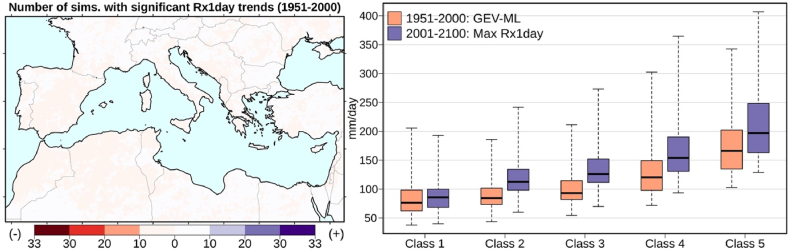
Number of simulations with significant (p-value < 0.05) positive (blue) and negative (red) non-parametric trends of annual daily precipitation extremes for 1951–2000 (left panel), and model-derived 100-year daily precipitation extremes based on a 1951–2000 GEV-ML fit and 2001–2100 absolute maximum values, for five precipitation classes (right panel). (Class 1: 0–200 mm/year, Class 2: 200–500 mm/year, Class 3: 500–1000 mm/year, Class 4: 1000–2000 mm/year, Class 5: >2000 mm/year). (For interpretation of the references to colour in this figure legend, the reader is referred to the Web version of this article.)

Throughout the region, the vast majority of the EURO-CORDEX simulations (30 out of 33) indicates insignificant trends of the annual daily rainfall extremes for the CTL period (Fig. 6 – left panel). Therefore, we derived the 100-year extremes of the past from the GEV-ML fitted distribution functions for this period. A comparison of these past 100-year extremes with the future 100-year extremes (absolute Rx1day maxima from the 2001–2100 projected time-series) for the five precipitation classes is shown in the right panel of Fig. 6 (EURO-CORDEX ensemble means). The median of Class 1 grid-cells (0–200 mm/year), indicates no apparent effect of climate change on the 100-year extremes. For the wetter regions (Classes 2–5), the 100-year extremes from the GEV-ML estimation (based on the 1951–2000 data) is approximately 20% lower than the 100-year extreme of the 2001–2100 projections. For example, the median of Class 5 grid cells daily extreme precipitation is 166 mm/day, whereas the 100-year extreme for the 21st century is 197 mm/d. The largest percentage difference (36%) is found for Class 3. The GEV-ML median value for the 100-year extreme is 93 mm/day, while the 21st-century projections suggest 126 mm/day.

**Fig. 7:**
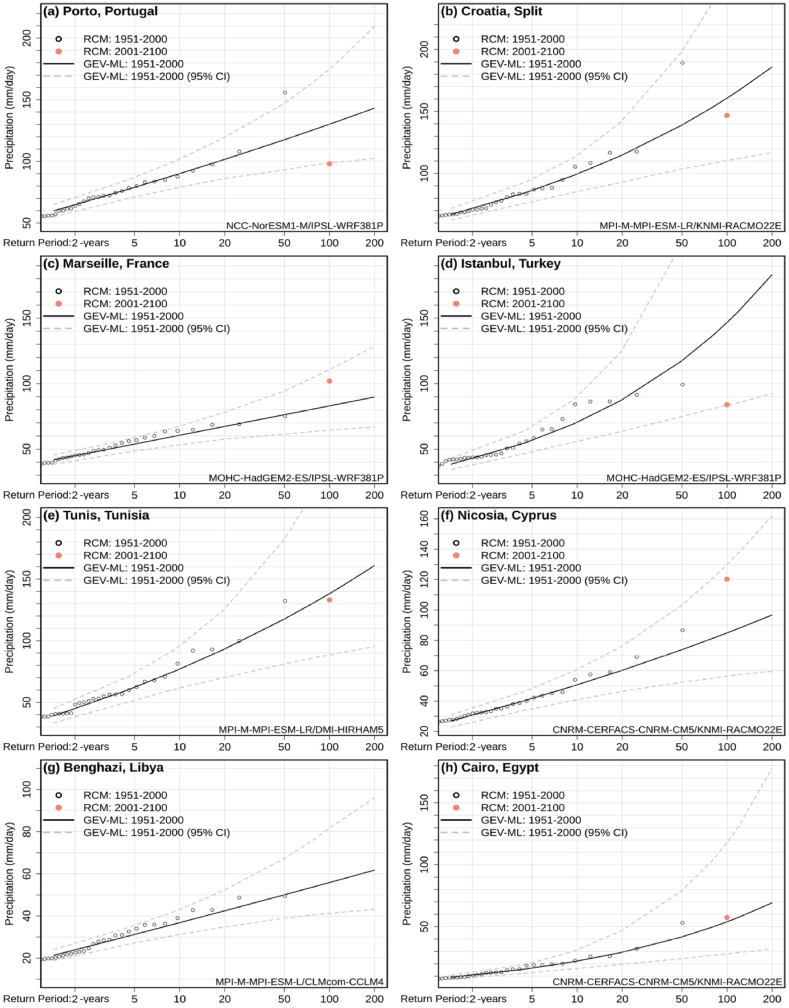
Extreme daily precipitation quantiles and 95% confidence intervals based on the GEV-ML method and the regional climate simulations for 1951–2000. The 100-year return period daily precipitation based on the 21st century projections are also shown (red circles). Results are presented for eight Mediterranean cities and the median-slope simulations. (For interpretation of the references to colour in this figure legend, the reader is referred to the Web version of this article.)

The 95% confidence intervals of the GEV-ML distributions, based on parametric bootstrapping with 1000 iterations, are illustrated in Fig. 7, for eight Mediterranean cities (representative for precipitation classes 1–4) for the simulation with the local median Rx1day Sen's Slope. For all cities, the absolute Rx1day extreme of the 2001–2100 period exceeds the GEV-ML-derived 100-year daily precipitation extreme (based on 1951–2000 data). For half of the tested cities (Porto, Marseille, Nicosia, and Benghazi), the 21st-century absolute extreme falls outside the 95% confidence interval, indicating a statistically significant change in the 100-year extremes.

**Fig. 8:**
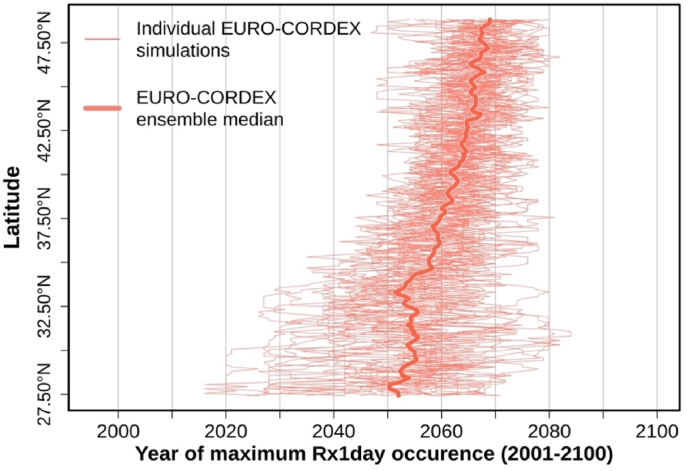
Projected year of the 100-year daily rainfall extreme (absolute-maximum Rx1day) occurrence with respect to the latitude according to the 33 EURO-CORDEX regional climate simulations.

Finally, we estimated the projected ‘year of occurrence’ of the 100-year daily extreme (maximum Rx1day) within the 21st century (Fig. 8). These 100-year extremes can occur at any time or any location within the Mediterranean, and individual climate projections can deviate significantly from this median estimation (not shown). Nevertheless, this analysis reveals an interesting pattern. For the southern Mediterranean latitudes (27.5–32.5 °N), the most severe events of the 21st century are more likely to occur earlier in the century, with the ensemble median of the EURO-CORDEX simulations occurring between 2050 and 2055. For northern Mediterranean regions (at latitudes >42.5 °N), the 100-year extremes are more likely to occur towards the latter part of the century, with the median between 2065 and 2070. Moreover, the spread of climate projections is considerably lower for the northern parts of the basin.

## 4 Discussion and conclusions

We have assessed the projected changes and 21st-century trends of extreme daily precipitation in the Mediterranean region by employing an ensemble of 33 regional climate simulations driven by a ‘business-as-usual’ pathway. Our results underscore the considerable uncertainty in the significance and direction of future projections. This uncertainty in representing precipitation extremes is to some degree expected because of the different parameterization methods of subgrid-scale processes used in the driving-global climate models and the regional simulations. This is also commonly found for dry climate regimes when various observational and reanalysis datasets are compared (Alexander et al., 2020; Zittis, 2018). Nonetheless, our analysis corroborates the north-south Mediterranean gradient in extreme precipitation trends. For the southern part of the basin and according to at least one-third of the models, significant decreasing trends in the magnitude of daily extremes are expected in the 21st century. The opposite (increasing trends) are projected by about 50% of the ensemble members for the northern part of the Mediterranean. This response to global warming agrees with other recent studies for the region (Conte et al., 2020; Lionello and Scarascia, 2020; Tramblay and Somot, 2018). Our results complement these previous analyses in several aspects. For example, we analyse regional climate models, known to outperform global models when it comes to extreme precipitation, and an unprecedented number of simulations (33), which provides additional insights when it comes to climate model uncertainties. Such uncertainties are quantified through inter-model ranges in the number of statistically significant trends. We also used the 33-member model ensemble to assess statistical significance of the changes in precipitation totals and extremes. Furthermore, by using 50- and 100-year periods, we allow for more robust statistical analysis of extreme precipitation quantile estimation.

At least for the drier parts of the Mediterranean, our findings for a ‘business-as-usual’ pathway propose the following paradox. Regardless of the overall decreasing trends of total precipitation, most of the region is projected to experience individual events of unprecedented magnitude at least once in the current century, with a larger likelihood of extremes during the second half (2051–2100). Such extremes will be at least 25% more intense for most of the region. For some of the arid parts of the domain, the absolute 50-year maximum daily precipitation magnitude is expected to double. The presented results indicate a future expansion of the precipitation paradox in the Mediterranean. This concept was introduced by Alpert et al. (2002) and referred to an observed increase of Mediterranean extreme daily rainfall despite the decrease in total annual rain. This can be partially attributed to a substantial change in the rainfall distribution, in which the ‘‘increase in variance’’ overcomes the ‘‘reduction in the mean’’ (Alpert et al., 2002; Meehl et al., 2000).

The results of our analysis of the EURO-CORDEX regional climate projections show that traditional methodologies for extreme-value analysis, as used for hydrological impact studies and infrastructure design, are challenged under global warming. The 1951–2000 GEV-ML estimations of the 100-year daily extremes are about 30% lower than the projected values for the 21st-century. This is evident mainly for the wetter parts of the Mediterranean region. Although this estimate is solely based on model data, it can provide useful insights on what kind of extreme events to expect in the future. Moreover, it justifies the need for climate change impact consideration in risk assessments and critical infrastructure design.

Hourly precipitation projections could be more informative to infer potential flood risk than daily data, especially for small catchments (<50 km2). Nevertheless, in the absence of publicly available, multi-model regional climate projections of such temporal resolution, the daily precipitation extremes can still provide useful insights. Excluding the effects of changes in land use and water management (for example, Tramblay et al., 2019), the projected increases in extreme daily rainfall could potentially be associated with unprecedented flooding events. Observed rainfall and flood records of the recent past have shown a mixed picture. Statistically significant increasing trends in annual daily rainfall extremes in the past 20 years have been found in the French Mediterranean (Ribes et al., 2019). For this region, Tramblay et al. (2019) found statistically significant increasing trends in the magnitude of flood events (above the 99th percentile) in 16 out of 171 basins and statistically significant negative trends in five basins, for 1970–2010. However, the number of flood events per year showed statistically significant decreasing trends for 45 basins and increasing trends for just one basin. For the northern rim of the Mediterranean region, Blöschl et al. (2019) found both increasing and decreasing trends in 7-day precipitation maxima (E-OBS data) for 1960–2010, but a majority of the trends was not statistically significant. These authors found that 13% out of 458 hydrometeorological stations showed statistically significant increasing trends in annual floods, whereas 31% showed statistically significant decreasing trends.

The contribution of extreme daily rainfall in the total annual budget is projected to increase throughout the region. This increase is expected to be strongest in north Africa and particularly in the Maghreb region. For this part of the domain there is high inter-model agreement (robustness) about substantial annual precipitation decreases.

According to several recent studies, there are strong indications that regional climate simulations at convection-permitting spatial resolution can improve the representation of extreme precipitation events (Zittis et al., 2017; Coppola et al., 2018 and references therein). Nevertheless, such numerical experiments are not widely applied for extended modelling domains and long-term climate simulations, due to their high computational cost. Extreme precipitation drivers, such as thermodynamics, atmospheric circulation and instability, and increasing sea surface temperatures, were not assessed in the present study. To have a more complete picture of future changes in extreme precipitation, these aspects should be considered through process-based studies. In addition, there is a need to integrate climate change projections with socio-economic scenarios to better understand and quantify any future changes in flood risks (Tramblay et al., 2019).

## Declaration of competing interest

The authors declare that they have no known competing financial interests or personal relationships that could have appeared to influence the work reported in this paper.
