# Supplementary material for: Revisiting future extreme precipitation trends in the Mediterranean
Source: Weather Clim Extrem. 2021 Dec;34:100380. doi: 10.1016/j.wace.2021.100380 (PMC8686183; doi:10.1016/j.wace.2021.100380)
Supplement: Multimedia component 1 [file mmc1.docx]

**SUPPLEMENTARY MATERIAL**

**Table S1.** List of EURO-CORDEX simulations considered in the present study.

|  | **GLOBAL MODEL** | **REGIONAL MODEL** |  |  | **GLOBAL MODEL** | **REGIONAL MODEL** |
| --- | --- | --- | --- | --- | --- | --- |
| **1.** | CNRM-CERFACS-CNRM-CM5 | CLMcom-CCLM4 |  | **18.** | MOHC-HadGEM2-ES | DMI-HIRHAM5 |
| **2.** | CNRM-CERFACS-CNRM-CM5 | CNRM-ALADIN |  | **19.** | MOHC-HadGEM2-ES | IPSL-WRF381P |
| **3.** | CNRM-CERFACS-CNRM-CM5 | DMI-HIRHAM5 |  | **20.** | MOHC-HadGEM2-ES | KNMI-RACMO22E |
| **4.** | CNRM-CERFACS-CNRM-CM5 | IPSL-WRF381P |  | **21.** | MOHC-HadGEM2-ES | MOHC-HadREM3 |
| **5.** | CNRM-CERFACS-CNRM-CM5 | KNMI-RACMO22E |  | **22.** | MPI-M-MPI-ESM-LR | CLMcom-CCLM4 |
| **6.** | CNRM-CERFACS-CNRM-CM5 | RMIB-UGent-ALARO |  | **23.** | MPI-M-MPI-ESM-LR | CLMcom-crCLIM |
| **7.** | ICHEC-EC-EARTH | CLMcom-CCLM4 |  | **24.** | MPI-M-MPI-ESM-LR | DMI-HIRHAM5 |
| **8.** | ICHEC-EC-EARTH | CLMcom-crCLIM |  | **25.** | MPI-M-MPI-ESM-LR | KNMI-RACMO22E |
| **9.** | ICHEC-EC-EARTH | DMI-HIRHAM5 |  | **26.** | MPI-M-MPI-ESM-LR | MOHC-HadREM3 |
| **10.** | ICHEC-EC-EARTH | MOHC-HadREM3 |  | **27.** | MPI-M-MPI-ESM-LR | MPI-CSC-REMO2009 |
| **11.** | ICHEC-EC-EARTH | KNMI-RACMO22E |  | **28.** | NCC-NorESM1-M | CLMcom-crCLIM |
| **12.** | IPSL-IPSL-CM5A-MR | DMI-HIRHAM5 |  | **29.** | NCC-NorESM1-M | DMI-HIRHAM5 |
| **13.** | IPSL-IPSL-CM5A-MR | IPSL-WRF381P |  | **30.** | NCC-NorESM1-M | IPSL-WRF381P |
| **14.** | IPSL-IPSL-CM5A-MR | KNMI-RACMO22E |  | **31.** | NCC-NorESM1-M | KNMI-RACMO22E |
| **15.** | IPSL-IPSL-CM5A-MR | MPI-CSC-REMO2009 |  | **32.** | NCC-NorESM1-M | MOHC-HadREM3 |
| **16.** | MOHC-HadGEM2-ES | CLMcom-CCLM4 |  | **33.** | NCC-NorESM1-M | MPI-CSC-REMO2009 |
| **17.** | MOHC-HadGEM2-ES | CLMcom-crCLIM |  |  |  |  |

**
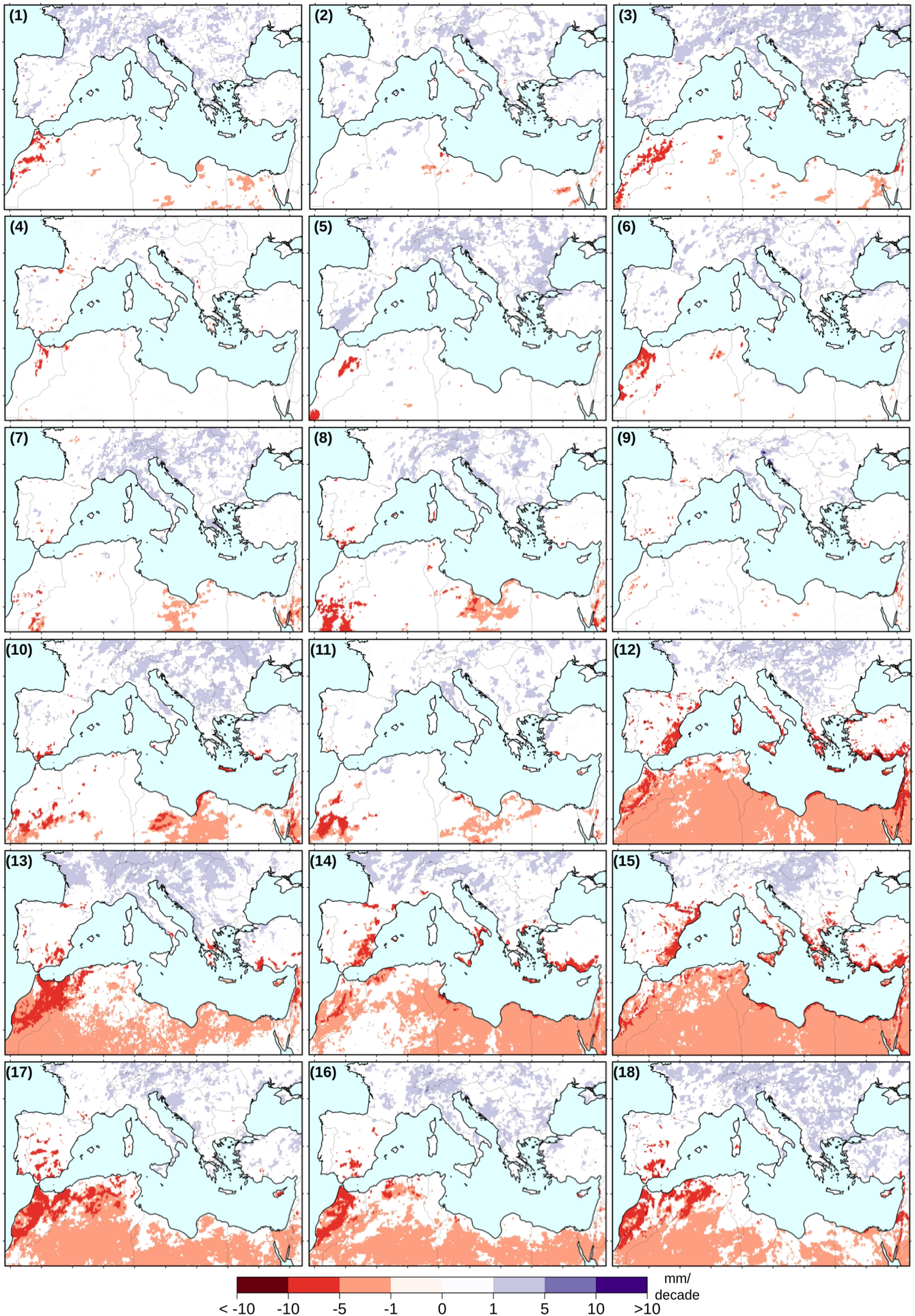
**

**Figure S1.** Individual model extreme daily precipitation (Rx1day) trends (Sen’s Slopes) in mm/decade for the 21^st^ century (2001-2100). Only statistically significant trends (Mann-Kendall test) are presented (See Table 1 for simulation IDs and details).


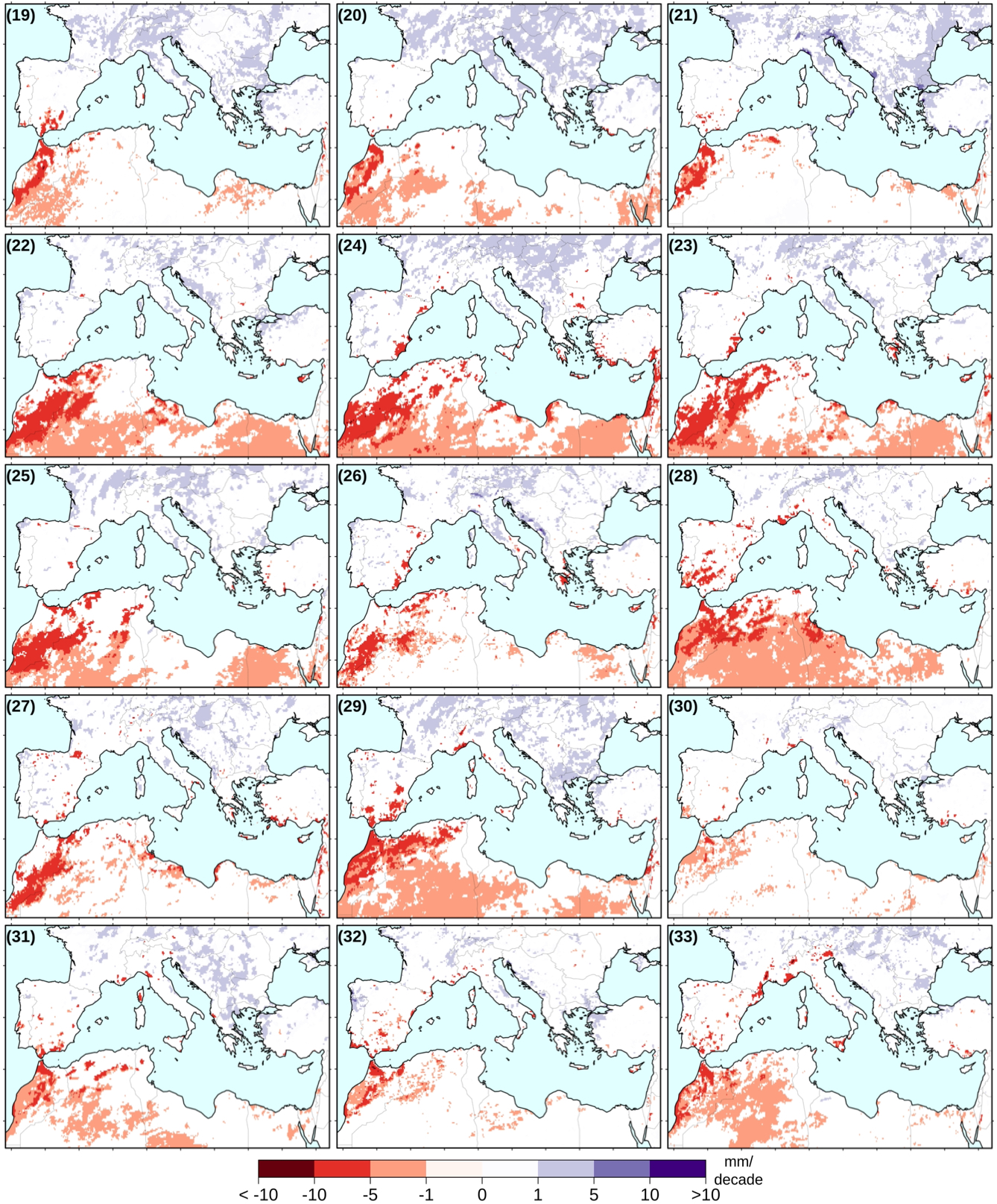


**Figure S1 (continued).** Individual model extreme daily precipitation (Rx1day) trends (Sen’s Slopes) in mm/decade for the 21^st^ century (2001-2100). Only statistically significant trends (Mann-Kendall test) are presented (See Table 1 for simulation IDs and details).
